# Dissecting the Binding Profile of PET Tracers to Corticobasal Degeneration Tau Fibrils

**DOI:** 10.1021/acschemneuro.1c00536

**Published:** 2021-08-31

**Authors:** Yang Zhou, Junhao Li, Agneta Nordberg, Hans Ågren

**Affiliations:** † Department of Physics and Astronomy, Uppsala University, Box 516, SE-751 20 Uppsala, Sweden; ‡ Division of Clinical Geriatrics, Center for Alzheimer Research, Department of Neurobiology, Care Sciences and Society, Karolinska Institutet, 141 84, Stockholm, Sweden; § Theme Aging Karolinska University Hospital, S-141 86 Stockholm, Sweden

**Keywords:** Corticobasal degeneration, metadynamics, positron emission tomography tracer, free energy surface, Alzheimer disease, binding profile

## Abstract

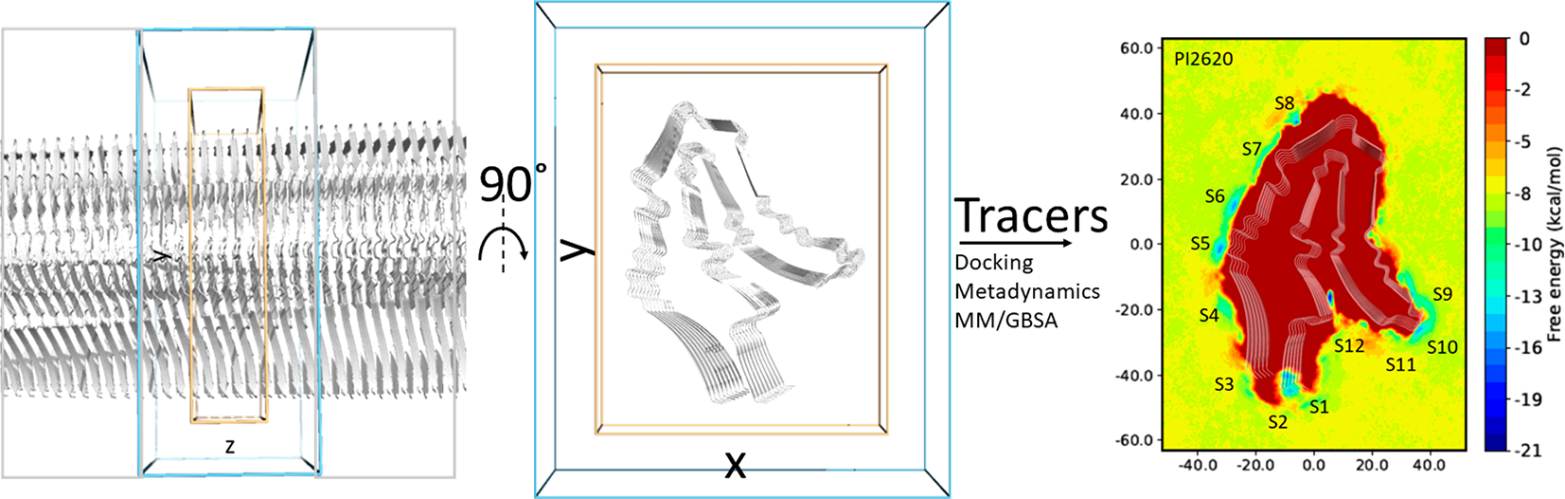

Alzheimer’s disease and primary tauopathies are characterized by the presence of tau pathology in brain. Several tau positron emission tomography (PET) tracers have been developed and studied in Alzheimer’s disease (AD), but there is still a lack of 4R-tau specific tracers for non-AD tauopathies. We here present the first computational study on the binding profiles of four tau different PET tracers, PI2620, CBD2115, PM-PBB3, and MK6240, to corticobasal degeneration (CBD) tau. The *in silico* results showed different preferences for the various binding sites on the 4R fibril, and especially an entry site, a concave site, and a core site showed high binding affinity to these tracers. The core site and entry site both showed higher binding affinity than the surface sites, but the tracers were less likely to enter these sites. PI2620, CBD2115, and PM-PBB3 all showed higher binding affinities to CBD tau than the 3R/4R tracer MK6240. The same strategy has also been applied to AD tau fibrils, and significant differences in selectivity of binding sites were also observed. A higher binding affinity was observed for CBD2115 and PM-PBB3 to AD tau compared to PI2620. None of the studied tracers showed a selectivity for 4R compared to 3R/4R tau. This study clearly shows that identified binding sites from cryo-EM with low resolution can be further refined by metadynamics simulations in order to provide atomic resolution of the binding modes as well as of the thermodynamic properties.

## Introduction

**Figure 1:**
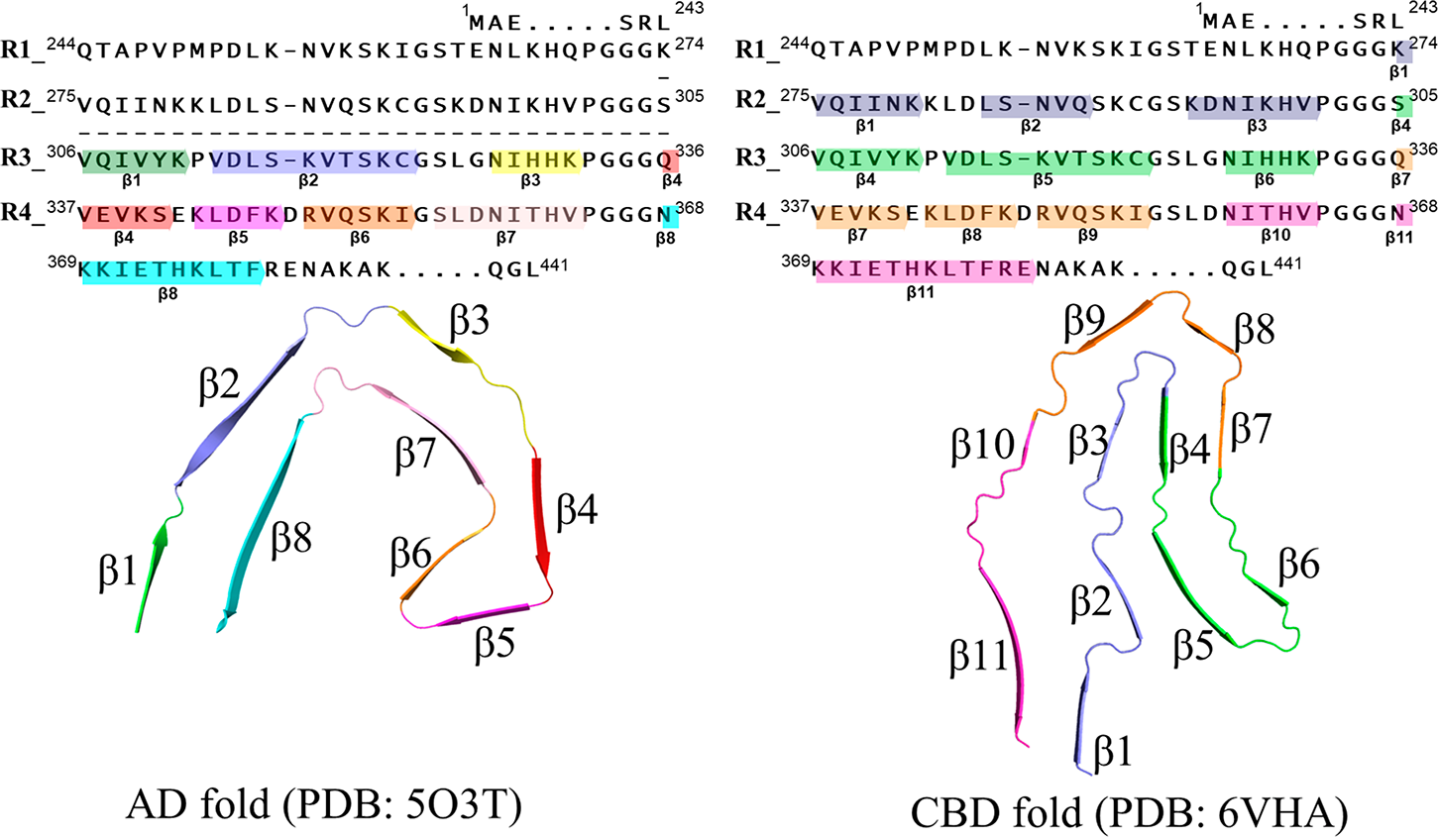
Illustration of the sequence and AD- and CBD-folding modes in the 4R-tau fibrils. The sequence alignment in the top two panels is adapted with permission from ref (29) (Copyright 2021, Elsevier) with new coloring style; the repeats 1–4 are abbreviated as R1–R4. The single chains from cryo-EM structures^28,29^ are illustrated in the bottom panel.

Several neurodegenerative disorders, named tauopathies, are characterized by tau protein aggregates in brain. There are, however, large histopathological differences between the tauopathies such as Alzheimer’s disease (AD) (neurofibrillary tangles) and primary tauopathies, such as progressive nuclear palsy (PSP), corticobasal degeneration (CBD), chronic traumatic encephalopathy (CTE), globular glial tauopathy (GGT), argyrophilic grain disease (AGD), and Pick’s disease (PiD).^1−4^ Tau aggregation has been identified as filaments, which generate abnormal tau fibrils in brain. The microtube-binding domain, enclosed either by three (3R) or four (4R) repeating subdomains, is folded to be β-sheet rich and is aggregated chain by chain in the formation of the tau fibrils.^5,6^ Components of such tau fibrils vary for different tauopathies, and while both 3R tau and 4R tau are present in AD and CTE, only 4R tau is found in CBD, GGT, AGD, and PSP, and 3R in PiD. Recent studies indicate that the tau fibril varies in its folding pattern between different tauopathies,^7^ indicating that each tauopathy is characterized by a disease-specific misfolding mode, e.g., AD-fold and CBD-fold (Figure 1).^8,9^ The imaging of the different tau fibrils has become an important goal for early detection and differential diagnosis of various neurodegenerative diseases.^8,10−12^

Several tau tracers have been developed for positron emission tomography (PET) imaging.^13^ [^18^F-]Flortaucipir (AV-1451 or T807),^14^ representing one of the first-generation tau PET tracer, was approved by FDA 2020 for discriminating AD from other non-AD dementia.^15^ Several second-generation tau PET tracers, including [^18^F-]MK6240,^16,17^ [^18^F-]RO-948,^18^ [^18^F-]PI2620,^19−21^ and [^18^F-]PM-PBB3,^22^ have shown high binding affinity in the brains of AD patients, and PI2620 and PM-PBB3 have also been reported to bind to tau in PSP^20,22^ and CBD^23^ patients. There is, however, a current lack of PET tracer molecules specifically binding to 4R tau.^16,24^ Recently, the cryoelectron microscopy (cryo-EM) structure^25^ of CBD tau was resolved^26^ while the structure of PSP tau still remains unknown. Compared with AD tau, the structure of the CBD tau fibril is very different (Figure 1),^27−29^ providing the possibility of designing selective tau probes targeting CBD. Despite the recently released cryo-EM structures for both AD and CBD tau, there are still several issues that need to be solved such as the structures of the tau-tracer complexes, the potential binding sites of the tracers in the tau fibril as well as the kinetics of the tracer binding to the fibril.^30−33^

In the present work we have analyzed the binding properties of four tau PET tracers aimed to be used for diagnostic purpose of different neurodegenerative diseases. Various computational methods representing different levels of theory were implemented to explore the potential tracer binding sites of CBD 4R tau (see Figure S1 for the workflow). First, the potential CBD tau binding sites of PI2620,^20^ PM-PBB3,^22^ CBD2115,^34^ and MK6240^17^ were studied by conventional molecular docking. Second, metadynamics simulations^35,36^ were applied to further explore the binding sites and the flexibility of the protein residues and to provide the possibility to sample the free energy surface of binding around the tau fibril surfaces. Furthermore, molecular mechanics/generalized Born and solvent accessibility (MM/GBSA)^37^ free energy calculations are used to estimate the binding free energies for all the identified binding sites. The binding modes of the tracers in the favorable binding sites were identified as well as the tracer-tau fibril interaction patterns. We also compared the binding properties of the tau tracers to 3R/4R AD tau. Our results provide atomistic insight into the binding profile of the different tracers, which may give valuable further guidance in the development of specific 4R tau PET tracers.

## Results

### Binding Sites of CBD-tau Found by Molecular Docking

**Figure 2:**
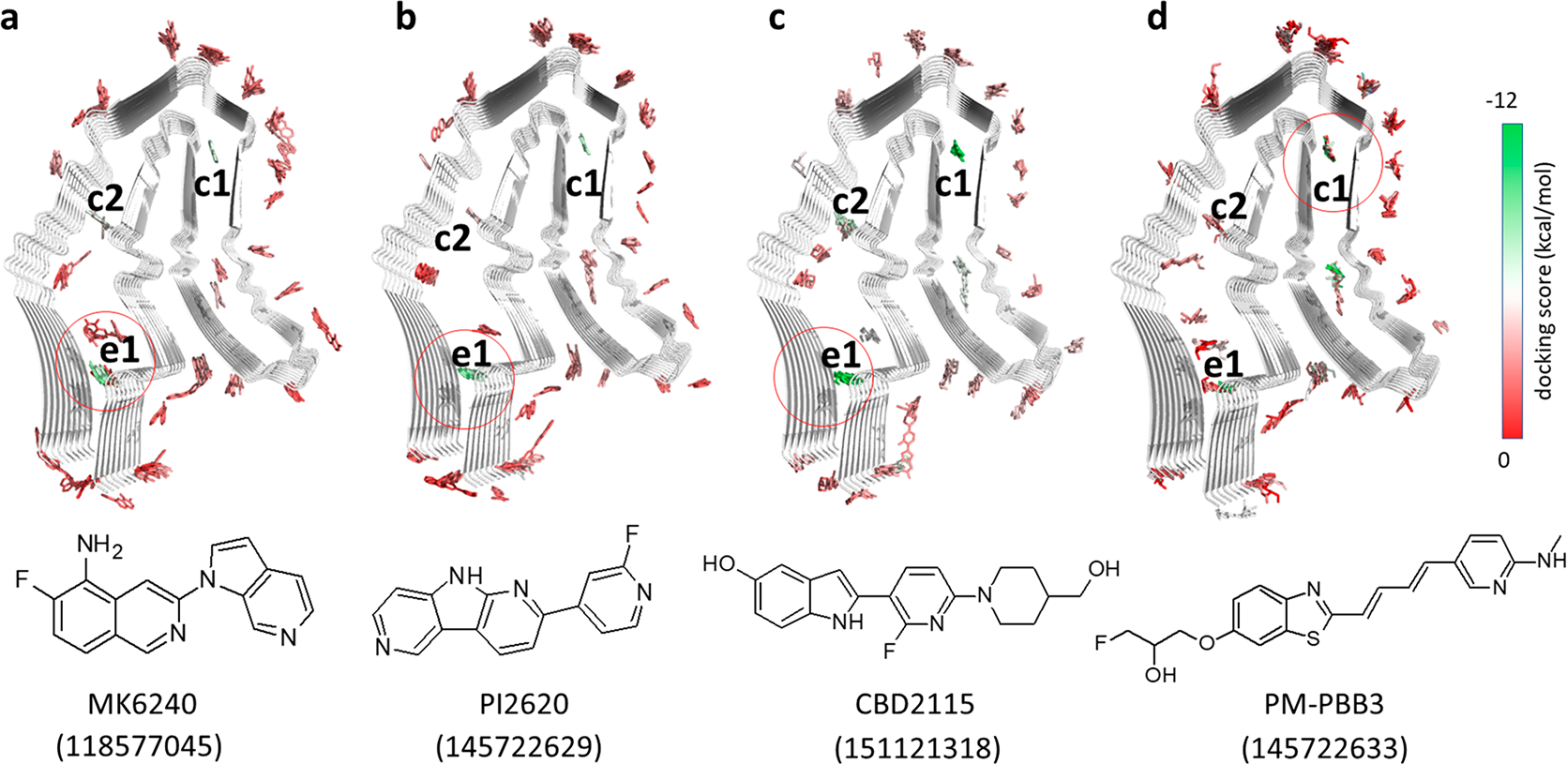
Docking profiles of tracers on CBD tau. The entry site and core sites 1 and 2 are abbreviated as e1, c1, and c2, respectively. The tracer at each site is colored according to the docking score. The tracer structures are shown. PubChem compound identifer (CID) of each tracer is shown in parentheses.

To profile the binding sites of CBD tau, we first carried out blind docking calculations for PI2620, CBD2115, PM-PBB3, and MK6240. The potential tracer binding sites predicted from the blind dockings are shown in Figure 2, where the tracers are highlighted with different colors corresponding to their docking scores. The docking results showed three binding sites, the entry site (e1) located between β1 and β11, the core site (c1) between β4 and β7, and the core site (c2) between β3 and β10 (see Figure 1 for the naming of β sheets), which were more favorable than other sites for the binding of the investigated tracers (Figure 2). Among these three sites, e1 was the most favorable site for all the tracers, in which the scores for CBD2115, PM-PBB3, PI2620, and MK6240 were −10.5 kcal/mol, −9.6 kcal/mol, −7.4 kcal/mol, and −7.3 kcal/mol, respectively (Table 1). The site c1 has more favorable docking scores than c2 (Table 1). In the molecular docking, the sites on the surface were docked with lower scores, generally lower than −6 kcal/mol, which are not favorable for binding. The docking scores of CBD2115 and PM-PBB3 (ranging from −6.4 to −10.5 kcal/mol) were in general better than those of PI2620 and MK6240 (ranging from −5.6 to −7.4 kcal/mol, Table 1), indicating the high binding affinities for CBD2115 and PM-PBB3 to the CBD tau.

**Table 1:** Docking Scores for the Tracers Binding at the c1, c2, and e1 Sites of CBD Tau (kcal/mol)

	c1	c2	e1
CBD2115	–9.6	–6.4	–10.5
PM-PBB3	–10.0	–7.3	–9.6
PI2620	–7.2	–5.6	–7.4
MK6240	–6.9	–5.6	–7.3

### Binding Sites of CBD-tau Predicted by Metadynamics Simulations

**Figure 3:**
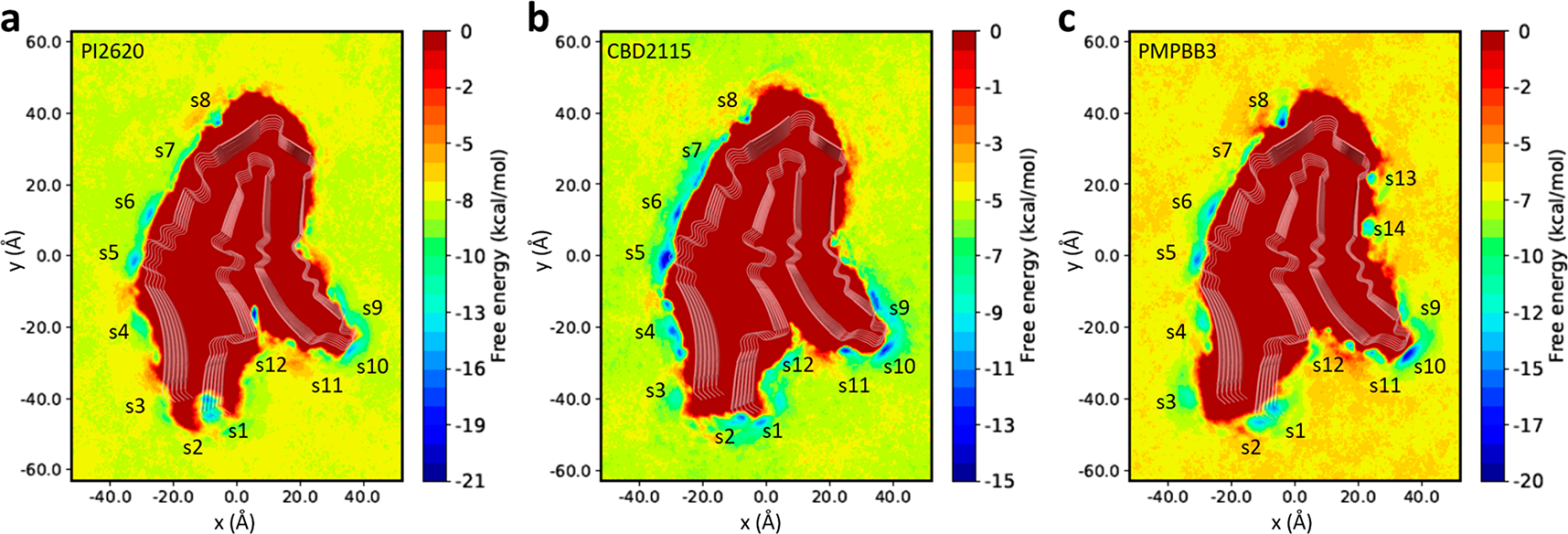
Free energy surfaces for the binding of tracers to AD tau derived from metadynamics simulations. (a) Free energy surface for the binding of PI2620. (b) Free energy surface for the binding of CBD2115. (c) Free energy surface for the binding of PM-PBB3. The identified binding sites are labeled with “s” and site identifier.

We further performed 3 μs metadynamics simulations for the CBD-tau fibril with each of the PI2620, CBD2115, and PM-PBB3 tracers (see Methods and Figure S2). The free energy surfaces (FES) for the tracer binding were calculated from these metadynamics simulations. As shown in Figure 3, PI2620, CBD2115, and PM-PBB3 demonstrated multiple free energy minima (marked from s1 to s14) on the surface of CBD tau. Some of the surface sites that were not favorable for binding in the docking studies became free energy minima in the FES, such as s3 and s4 near β11, s5 and s6 near the loop between β11 and β10, s7 and s8 near the loop between β10 and β9, and s9, s10, and s11 between the loops near β6 and β5 (Figure 3). PI2620 showed two minima s1 and s2 between β11 and β1, which were found to be closer to β1 for CBD2115. PI2620, but not CBD2115, demonstrates a deep free energy minimum between β5 and β2 (see s12 in Figure 3a). Two free energy minima near β7 (s13 and s14) only show up in the PM-PBB3 system.

The differences in free energies between the tracers in solvent and on the surface of tau were also calculated as approximated binding free energies (Table S1). Most of the binding sites were associated with a moderate binding free energy for the tracers. Three of the PI2620 binding sites (s1, s6, and s12) showed a binding free energy below −7 kcal/mol, in which the binding free energy for s12 was −11.4 kcal/mol, indicating that s12 is a very favorable site for PI2620 binding. Only one site exhibited a free energy below −7 kcal/mol (s5, −7.7 kcal/mol) for CBD2115. Nevertheless, for PM-PBB3, five low binding-free-energy binding sites were found, in which s1 and s6 are close to those in PI2620 and s5 is close to those in CBD2115. The sites s8 and s10 have stronger binding affinities for PM-PBB3 (−10.2 and −9.3 kcal/mol, respectively) than the other two tracers. PI2620 seems to possess some less favorable sites with free energy between −3 kcal/mol and −1 kcal/mol. Most of the sites for CBD2115 show moderate binding affinity between −5 kcal/mol to −8 kcal/mol, in which s5 was the most favorable site (−7.7 kcal/mol). Two sites (s1 and s2) close to the entry region were observed for PI2620 but not as deep as e1 identified in the docking study.

### Comparison with Docking and Metadynamics Simulation Results on AD-tau

We also docked MK6240, CBD2115, PI2620, and PM-PBB3 to the entry site (E1) and surface site (S4) with the highest binding free energy identified in the previous study on AD-tau.^38^ The results are shown in Table S2. At the E1 position, the scores of MK6240, PI2620, CBD2115, and PM-PBB3 were −9.2 kcal/mol, −8.8 kcal/mol, −8.5 kcal/mol, and −10.6 kcal/mol, respectively. It is notable that the docking score for the binding of MK6240 at the E1 site of AD tau showed stronger binding affinity than the e1 site in CBD tau (−9.2 versus −7.2 kcal/mol; see Table S2 and Table 1).

**Figure 4:**
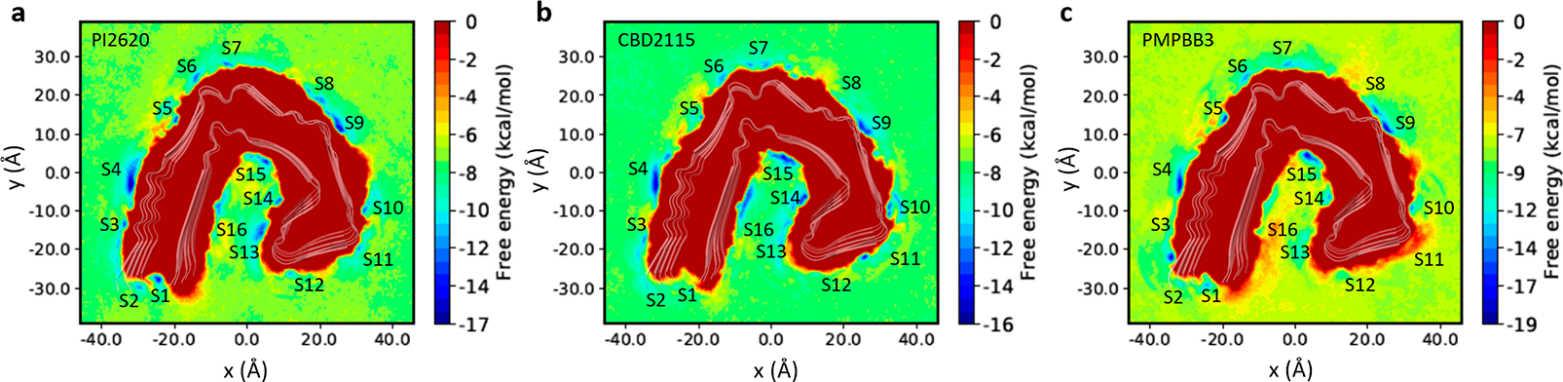
Free energy surfaces for the binding of tracers to AD tau derived from metadynamics simulations. (a) Free energy surfaces for the binding of PI2620. (b) Free energy surface for the binding of CBD2115. (c) Free energy surface for the binding of PM-PBB3. The identified binding sites are labeled with “S” and site identifier.

We further applied the same metadynamics simulation strategy on AD-tau for the other three tracers (Figure S3). Multiple free energy minima (marked from S1 to S16) were also found on the FES as shown in Figure 4. The concave binding sites (marked as V1–V3 in the previous study) and the entry site E1 of AD tau were identified as local minima in our simulations and denoted as S13–S15 and S1, respectively.^31^ This indicates that the surface sites determined in the previous study can be reidentified in this work using different collective variables (CVs). Most of the free energy minima were commonly found for all three tracers, such as S3 and S4 near the loop between β1 and β2, S5 close to β2, S6 and S7 near the loop between β2 and β3. By comparing the free energy of these energy minima (Table S3), we found that S1 and S15 were favorable for PI2620 with the free energy of −6.3 kcal/mol and −6.5 kcal/mol, respectively. For the CBD2115 tracer, S4, S11, and S15 were favorable sites with the free energies of −7.9 kcal/mol, −6.7 kcal/mol, and −6.2 kcal/mol, respectively. PM-PBB3 showed low binding affinity to five sites with the free energy lower than −6 kcal/mol, in which S1 corresponds to the entry site according to our previous study.^38^ The sites S11 and S15 have also been identified as the surface site in a previous binding site study on MK6240 and T807.^31^

### Free Energy Calculations by MM/GBSA

To compare the affinity of the binding site obtained in the docking to those found in the metadynamics simulations, we calculated the MM/GBSA binding free energy for the sites found in the docking study and the metadynamics simulations (Table 2). As presented in Table 2, some binding values differed between the docking and metadynamics studies. For example, in the docking studies, c2 was the least favorable site among the three sites (Table 1). In the MM/GBSA calculations, the binding free energies at c2 were more favorable than c1 for PI2620 (−38.4 versus −34.9 kcal/mol) and MK6240 (−42.4 versus −20.0 kcal/mol, Table 2). PM-PBB3 showed more favorable binding sites than other tracers, such as c1 (−55.2 kcal/mol), c2 (−52.9 kcal/mol), e1 (−43.9 kcal/mol), and s1 (−43.9 kcal/mol).

**Table 2:** MM/GBSA Binding Free Energies of the Tracers to CBD and AD Tau at Different Sites^a^

	sites^b^	MK6240	PI2620	CBD2115	PM-PBB3
CBD tau	c1	–20.0	–34.9	**-57.8**	**-55.2**
	c2	**-42.4**^c^	–38.4	–39.2	**-52.9**
	e1	–30.5	**-41.5**	**-71.0**	**-66.3**
	s1	-d	–19.2	**-43.9**	**-43.9**
	s5	-	–22.3	–33.0	-
	s6	-	–22.3	–37.7	-
	s12	-	**-46.8**	-	–29.1
AD tau	S1	**-60.7**	**-51.2**	**-87.0**	**-113.9**
	S4	–36.5	**-41.2**	**-50.5**	**-32.4**

a All terms are in kcal/mol and calculated from 1000 snapshots (90–100 ns). The standard errors are in the range of 0.5–2.0 kcal/mol.

b Names of sites for CBD tau start with lower case letters: c1, c2, and e1 for docking identified sites, and s1, s5, s6, and s12 for metadynamics identified sites. Names of sites for AD tau start with upper case letters: S1 and S4 for metadynamics identified sites.

c Sites with strong binding affinities are shown in bolded text.

d No binding pose (from docking) was detected for the corresponding site.

From the MM/GBSA calculations, MK6240 seems to bind to AD-tau (S1, −60.7 kcal/mol) with stronger affinity than to CBD tau (c2, −42.4 kcal/mol). At the e1 site of CBD-tau, the MM/GBSA binding free energy for CBD2115 (−71.0 kcal/mol) is significantly better than that for PI2620 (−41.5 kcal/mol) and MK6240 (−30.5 kcal/mol). The core site c1 was also quite favorable for CBD2115 with the binding free energy of −57.8 kcal/mol, compared to PI2620 (−34.9 kcal/mol) and MK6240 (−20.0 kcal/mol). This demonstrates that CBD2115 showed a more favorable binding with CBD-tau than PI2620. Furthermore, CBD2115 strongly binds to site S1 of AD-tau with the binding free energy of −87.0 kcal/mol, while both MK6240 and PI2620 showed lower binding free energy to the S1 site (−60.7 kcal/mol and −51.2 kcal/mol, respectively). We note here that the differences in the absolute values of docking scores and MM/GBSA results are mainly caused by the solvation effect being implicitly included in MM/GBSA, making the MM/GBSA absolute values larger than the Glide docking scores. Thus, differential energies should be compared between the two methods, not absolute energies.

The two favorable CBD-tau binding sites for PI2620, i.e., entry site e1 and surface site s12, showed binding free energies lower than −40 kcal/mol. In a previous study PI2620 demonstrated higher binding affinity to the core site and entry site compared to the concave sites of the AD-tau fibrils.^38^ Similarly, for CBD-tau, the entry site e1 showed a binding free energy of −41.5 kcal/mol while site s12 at the concave region showed a stronger binding affinity to PI2620 than that at the core site.

### Binding Mode Analysis

**Figure 5:**
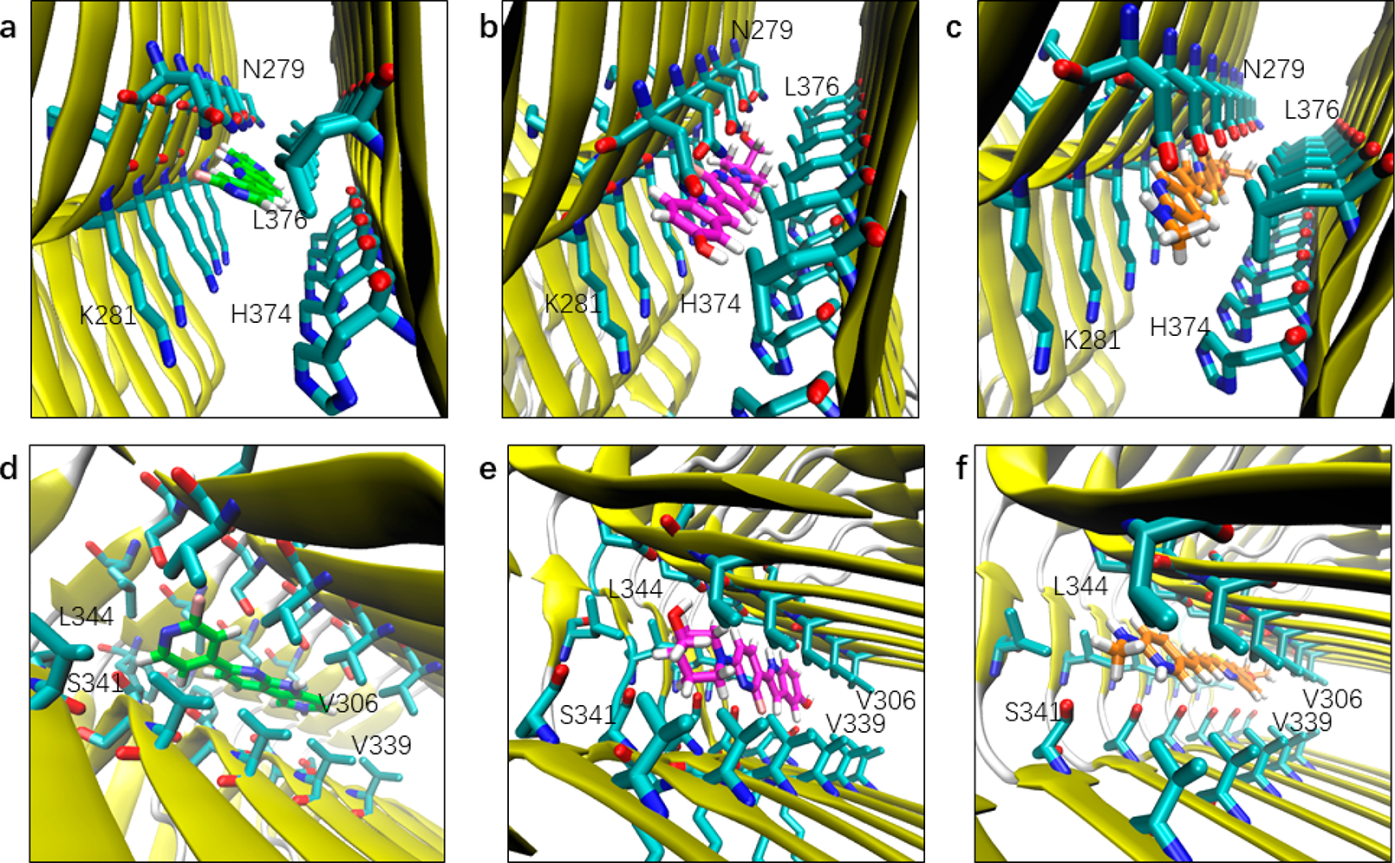
Binding mode of tracers at the entry and core sites of CBD tau. (a) Binding mode of PI2620 at e1. (b) Binding mode of CBD2115 at e1. (c) Binding mode of PM-PBB3 at e1. (d) Binding mode of PI2620 at c1. (e) Binding mode of CBD2115 at c1. (f) Binding mode of PM-PBB3 at c1. The heavy atoms of residues within 5 Å of the tracer heavy atoms are displayed.

**Figure 6:**
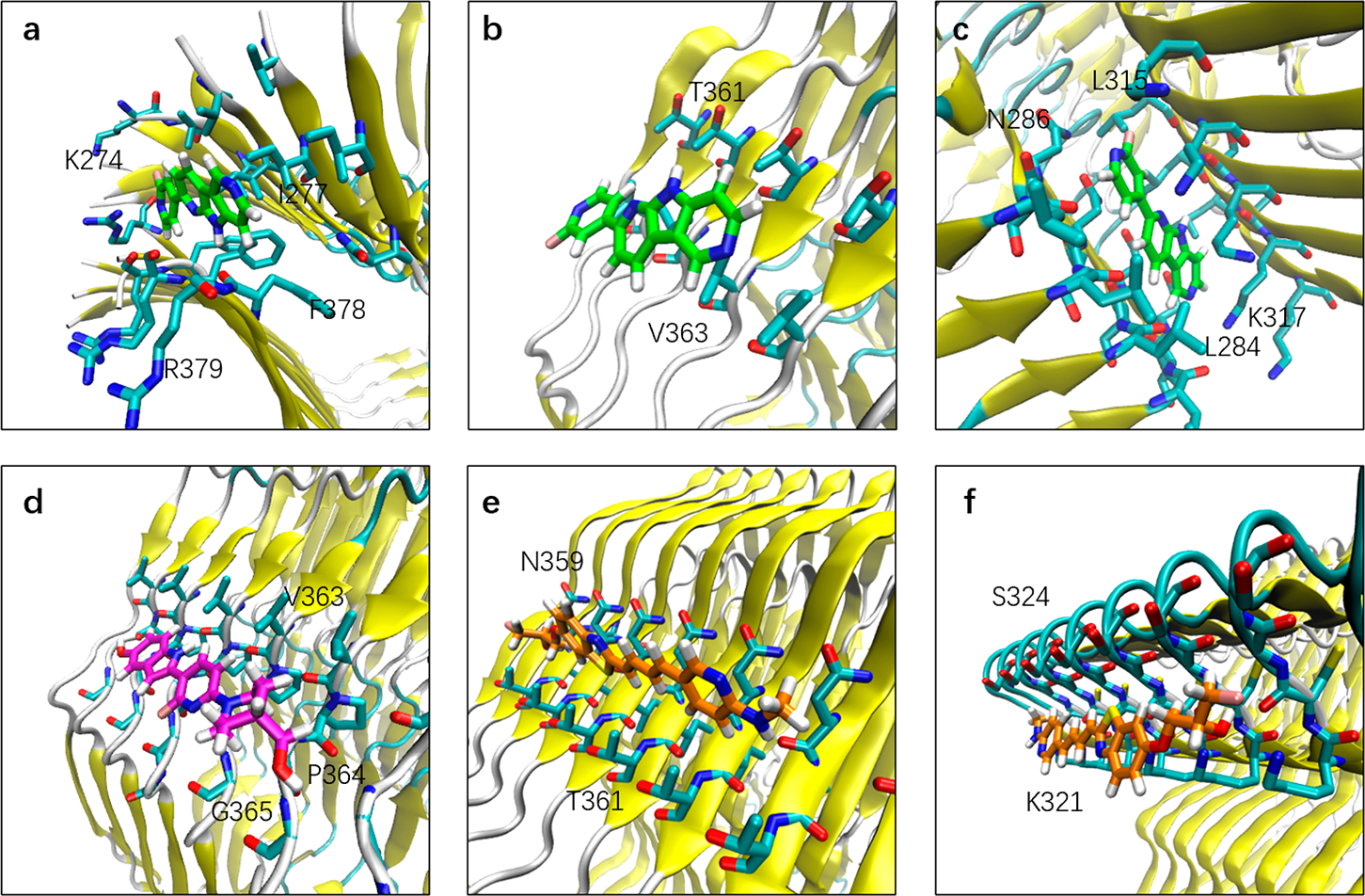
Binding mode of tracers at the surface sites of CBD tau. (a) Binding mode of PI2620 at s1. (b) Binding mode of PI2620 at s5. (c) Binding mode of PI2620 at s12. (d) Binding mode of CBD2115 at s6. (e) Binding mode of PM-PBB3 at s8. (f) Binding mode of PM-PBB3 at s10. The heavy atoms of residues within 5 Å of the tracer heavy atoms are displayed.

The binding modes of the tracers in the core sites and surface sites are shown in Figures 5 and 6, respectively. PI2620 demonstrated slightly different binding properties compared to both CBD2115 and PM-PBB3. Core site c1 and e1 appeared to be more favorable for CBD2115 and PM-PBB3 while less favorable for PI2620 (Table 2). For PI2620, there is a certain angle between the pyridine and dipyridine rings, which do not allow a linear configuration as in PM-PBB3 and CBD2115. The linear structures of CBD2115 and PM-PBB3 seemed to be superior for binding compared to PI2620 in the narrow and long hydrophobic region composed of Val306, Val339, Leu344 such as c1 and in the region composed of Asn296, Lys294, and His362 such as c2 (Figure 5).

From the structural point of view, the surface areas of CBD2115 and PM-PBB3 are larger, leading to a larger contact surface between CBD2115/PM-PBB3 and the fibril. As shown in Figure 6, CBD2115 interacts with about five chains of the fibril, PM-PBB3 interacts with about six chains, while PI2620 interacts with only four chains. The larger surface of CBD2115 enables a stronger binding in the hydrophobic environment in the core sites and in the entry site. At the e1 site, the aromatic and aliphatic rings of CBD2115 are buried in a hydrophobic environment composed of the side chain of Lys281 and Leu376. In addition, the hydroxyl groups on the piperidine ring and the indole ring of CBD2115 can form hydrogen bond interactions with His374 (7.2% of occupancy), Lys280 (16.5% of occupancy), and Asn279 (8.9% of occupancy) of the fibril (Figure S4), increasing the binding ability of CBD2115 at the e1 site. Compared with CBD2115, PI2620 lacks the interaction between these two hydroxyl groups and polar groups. Therefore, the docking score and MM/GBSA binding free energy of CBD2115 seem to be more favorable than those of PMPBB3 and PI2620. However, the larger surface of CBD2115 on the surface sites results in a solvent exposure, which is not favorable for binding. At the s5 site, CBD2115 was attached to the hydrophobic area formed by Val363 and Gly365, which is not conducive to the binding.

In s12, PI2620 was wrapped in the hydrophobic cavity between β2 and β5. This site corresponded to the concave form of AD-tau but was narrower. It was formed by the fatty chains of Leu284, Leu315, and Lys317 (Figure 6) and was induced-fitted to the tracer. In addition, the nitrogen on the PI2620 pyridine was close to the inner Asn286 at s12, forming a polar interaction. Such favorable interactions enhance the binding affinity of PI2620 to site s12, giving the MM/GBSA free energy as high as −46.8 kcal/mol, which is larger than that at core c1 (−34.9 kcal/mol) and c2 (−38.4 kcal/mol).

At the surface sites, PI2620 is only weakly attached to the residues and is largely exposed to the solvent (Figure 6b). For CBD2115, the situation was similar. The most favorable surface site of CBD2115 was s6. At this site, CBD2115 was attached to the shallow pocket formed by Val363, Pro364, and Gly365 (Figure 6d). On the surface sites, the hydrophobic aromatic ring structures of CBD2115 and PM-PBB3 were exposed to the solvent, which is detrimental to the binding affinity.

## Discussion

*In vitro* binding data for CBD2115, PM-PBB3, PI2640, and MK6240 in postmortem cortical brain tissue from AD, PSP, or CBD subjects indicate differences in binding properties between the different tau tracers.^17,34,39,40^ [^3^H-]MK6240 has shown a 100 times higher affinity in AD brain tissue compared to both PSP and CBD brain tissue.^33^ 10 and 2 times lower *K*_d_ binding values in PSP and CBD tissues have been reported for [^3^H-]CBD2115 compared to [^3^H-]MK6240.^34^ The same authors, Lindberg et al., also observed a 5 times lower *K*_d_ value for [^3^H-]CBD2115 in PSP compared to CBD brain tissues.^34^ For [^18^F-]PI2620, comparable *K*_d_ values have been reported for AD and PSP,^41^ as well as for [^18^F-]PM-PBB3 in AD and PSP tissue.^22^

PET studies with [^18^F-]PI6240, [^18^F-]MK6240, and [^18^F-]PM-PBB3 have been reported in the literature for the AD, PSP, and CBD patients.^17,19−22,39,41^ These studies clearly demonstrate that MK6240 shows no significant binding in PSP and CBD patients and therefore can discriminate AD from other tauopathies by its selective binding in the brain of AD patients.^17^ This observation is in agreement with the *in vitro* binding studies discussed above and is also the reason why in the present study MK6240 was not selected for the metadynamics simulations for CBD tau. PI6240 and PM-PBB3 both show higher binding in brain of AD compared to CBD and PSP, and the regional distribution in brain differs also between AD and the primary tauopathies PSP, CBD.^20,22,42^ CBD-2115 is an experimental compound designed as a specific 4R tracer for primary tauopathies, but since it also shows high affinity binding to AD tissue as well as *in vivo* observation of low uptake in mice and non-human primates,^34^ it is less likely to be further translated as a 4R tau PET tracer for studies in man.^34^

The score of MK6240 in the docking to AD tau was higher than that of CBD tau and also suggested that MK6240 might be more favorable than PI2620 and CBD2115 for AD tau. This finding is in agreement with the *in vitro* binding data in postmortem AD brain tissue demonstrating two binding sites with IC_50_ values of 1 pM (58% of the binding sites) and 12 nM,^39^ as well as the *in vivo* PET studies.^17,43^ The selective binding of MK6240 to AD tau fibrils is well explained by the unraveled atomistic interaction with two favorable sites in AD tau identified for MK6240.

Molecular docking provides preliminary information about the potential binding sites of CBD tau. However, the protein is treated as a rigid receptor in such dockings. In previous studies on AD tau, we successfully used metadynamics to identify high potential binding sites that cannot be found by molecular docking.^31,38^ In the present study, we followed the methodology of metadynamics for finding the binding sites of the fibril with new settings of collective variables. Unlike the previous studies,^31,38^ which used polar coordinates (*d* and χ) of the fibril cross-section as CVs, we used in the present study the Cartesian coordinates (*x* and *y*) of the cross-section (Figure S5). These CVs enable us to associate the free energy minima with the binding sites more straightforwardly. The differing of results from those of docking is most probably caused by the inclusion of the flexibility of the fibrils and the ligand-induced fit effect. In fact, neither entry site nor core sites identified in the docking study were found on the free energy surfaces. In our simulations, the chain was duplicated multiple times and applied in a periodic boundary condition. By this way, we could use a few chains to mimic the structure of a long fibril which consists of hundreds of chains, and the number of chains could be restricted in the fibril framework in the simulations. However, due to the use of the periodic boundary condition, the two ends of the fibril were not exposed for the tracers to enter. Although the “entry” region on the side of the fibril is exposed to the solvent, the metadynamics simulations did not predict the tracers to enter at e1, c1, or c2 via this region. The framework thus restricts the chains so that the structure cannot open to let tracers enter, which is closer to the situation of the real state of the fibrils.

Docking and metadynamics simulations provide a complementary picture of the potential binding sites of the fibrils. However, the binding free energies from MM/GBSA are clearly different from the docking scores, because the protein flexibility and solvent effect are included in MM/GBSA calculations. The results, indicate that the binding free energies for CBD2115 are generally more favorable for both CBD-tau and AD-tau. The MM/GBSA results also show the preference of MK6240 and PM-PBB3 to AD-tau. The metadynamics predicted sites S14 and S15 are also in line with those sites identified in a very recent cryo-EM study of the PM-PBB3 binding to the AD-tau fibril, in which S14 and S15 can overlap with the experimental detected major sites 2a, 2b, and 3 (Figure S6).^40^ The identified binding sites from cryo-EM with low resolution can thus be further refined by metadynamics to provide atomic resolution of the binding modes as well as of the thermodynamic properties. In the metadynamics simulations, several surface sites are identified on the surface of CBD-tau (s3–s12). Therefore, by use of metadynamics, the surface sites are located in free energy minima, while in the docking studies none of the surface sites actually show a favorable score. However, the MM/GBSA binding free energies of the surface sites of CBD-tau are also not as favorable as the entry site, core sites, or concave site. The most favorable surface sites for PI2620 on CBD tau seem to be s5 and s6. It is notable that the concave site s12 is more accessible than the core sites, which can only be approached from the two ends of the fibril. This may imply that this site is more suitable for binding. Thus, site s12 is worthwhile to consider for structural optimization of PI2620, although this site was not found favorable for CBD2115.

For the high-affinity binding sites, we analyzed the binding modes of tracers to tau fibril and identified the key interactions. Our findings clearly show that due to the structural differences between the AD and CBD tau fibrils, the same tracer can demonstrate different binding characteristics for the two tau fibrils. None of the four tracers showed a higher preference for the binding sites on CBD compared to the AD tau fibril. CBD2115 was designed as a tentative 4R tracer but is predicted by the present *in silico* calculations as well as demonstrated in recent *in vitro* tissue binding studies^34^ to show high affinity binding to the AD tau fibrils. Interestingly, we were able to demonstrate that the different tau tracers preferred different binding sites at the same CBD tau fibril. PI2620 showed the strongest binding affinity to CBD-tau at the concave site, here named s12, which was not favorable for CBD2115. In contrast CBD2115 demonstrated a stronger binding affinity to the entry site e1 and core site c1, with e1 being the most favorable site (Table 2). PM-PBB3 also showed a high affinity to the entry site e1.

Through the computational results we were able to localize the binding sites and thereby also perform determination of the binding strengths in terms of interactions (e.g., polar, hydrophilic, van der Waals, hydrophilic interactions) and in terms of structural motifs (residue sequence and atomic composition and the folding form of the fibrils). We hereby underline the importance of the induced fit mechanism where tracer and fibril mutually and dynamically perturb the structures of the counterpart. For example, the root-mean-square deviation (RMSD) values for the Cα atoms are affected differently by different tracers (Figure S7). We also observed that the π–π stacking contributes more to the binding free energy than other interactions during the course of MD simulations. For example, PI2626 forms stable π–π interaction to the Asn279 and Lys281 at the e1 site of CBD tau (Figure S8). These results can lead to further understanding of structure–property relationships and so form the basis for a further design of tracers with improved performance and specificity.

In the present study we demonstrated multiple binding sites for PI2640, PM-PBB3, CBD2215 on the CBD tau fibril and the binding sites for each tracer varied as well. For the same tracer, the binding sites identified using different modeling approaches were slightly different. Overall, since the tracers can be buried, the core site and the entry site showed higher binding affinity than the surface sites, but at the same time the tracer was less likely to enter these sites. From the MM/GBSA calculations we found that PI2620, CBD2115, and PM-PBB3 showed higher binding affinities to CBD tau than MK6240. CBD2115 and PM-PBB3 showed some advantages over PI2620 in reaching their binding sites due to the linear scaffold, and by calculations, we also showed that the binding affinity for CBD2115 and PM-PBB3 to AD tau was higher than for PI2620.

Although several components play a role for a successful molecular tracer, including kinetics, transition state barriers, binding pocket residence times^44,45^ in *in vitro* studies, and blood–brain barrier penetration, lipophilicity and plasma or membrane protein binding in *in vivo* studies, the ultimate potency of the tracer depends on the tracer–fibril binding strength.^4,46^ That in turn is delicately dependent on atomic structure, both of the tracer and the receiving protein fibril. The application of a consistent hierarchical multiple-level approach representing different levels of rigor and efficiency as presented in this work can make it possible to understand the binding mechanism of PET tracers such as PI2620, CBD2115, and PM-PBB3. We can conclude from our studies that none of the tracers showed selective specificity for 4R tau in primary tauopathies. We are convinced that the binding characteristics for four tracers to CBD tau and AD tau fibrils of AD will be useful for the further development of new tracers with improved binding affinity and high selectivity targeting specifically 4R tau fibrils. Work along these lines is ongoing.

## Methods

### System Preparation and Molecular Docking

The structures of the CBD tau fibril (PDB code 6VHA)^29^ and AD tau fibril (PDB code 5O3T)^28^ were retrieved from the Protein Data Bank (http://www.rcsb.org/) and was prepared using the Protein Preparation Wizard module in the Schrödinger Suite.^47^ The protonation states of CBD tau were assigned for histidine 299, 330, and 374 at the ε position, 362 at the δ position, and 329 at both δ and ε positions, and AD tau for histidine 362 and 374 at the ε position, 330 at the δ position, and 329 at both δ and ε positions. The cofactor in CBD tau fibril was not modeled. It is possible for tracers to bind to fuzzy coats,^48^ which are flexible and can cover the surface sites of tau; in this case, the fuzzy coat region was removed in the simulations. The number of fibril chains should be sufficient to cover the tracers along the *z*-axis for molecular dynamics (MD) simulations. To create a multichain fibril, the first chain in the cryo-EM structure was superimposed to the last chain, followed by the removal of one of the overlapping chains. According to the size of a single chain and the rotational angles of the fibril, we kept nine chains for the CBD tau and five chains for the AD tau in all the simulations.

The initial structures of the tracer molecules were obtained from PubChem (https://pubchem.ncbi.nlm.nih.gov/) and prepared by the LigPrep module in Schrödinger Suite (version 2019-1). The Glide^47,49,50^ module was used for molecular docking. In order to search the potential regions for tracer binding, the center of Glide grid box was generated by gridding the space of the whole fibril with the spacing interval of 5 Å and the radius of 18 Å. The dockings were performed with standard precision (SP) mode with default settings.

### Metadynamics Simulations

Well-tempered metadynamics simulations were carried out using the PLUMED (version 2.5.0) patched GROMACS (version 2018.1) for both AD and CBD tau.^51,52^ The tracer molecule was randomly placed in the solvent as a starting complex. The Amber ff99SB-ildn force field^53^ was used for the protein and the general Amber force field^54^ for tracers. The partial charges of the inhibitors were calculated through the restrained electrostatic potential (RESP) fitting procedure,^55^ in which the electrostatic potential points were generated by Gaussian 09 (version D01)^56^ with the calculations carried out at the Hartree–Fock level using the 6-31G* basis set. The TIP3P water model^57^ was used to solvate a fibril-tracer system. The counterions were added to neutralize the system, and the ionic concentration was set to 0.15 M by adding Na^+^ and Cl^–^ ions. Energy minimization and restrained equilibration simulations in the *NVT* ensemble (*T* = 300 K, 100 ps) and *NPT* ensemble (*T* = 300 K, *P* = 1 atm, 100 ps) were conducted. Before the metadynamics simulations, each system was additionally equilibrated under the *NPT* ensemble for 100 ns without any constraints.

For each metadynamics production simulation, two collective variables (CVs) were used to describe the position of the tracer relative to the fibril (Figure S5), which are (1) the *x* coordinate of the center of mass of the tracer (CV1) and (2) the *y* coordinate of the center of mass of the tracer (CV2). To limit the sampling space, the *z* coordinate of the tracer was restrained from −5 Å to 5 Å with harmonic potential. The initial Gaussian height was set as 0.2 kcal/mol with a bias factor of 6 and the temperature at 300 K.^58^ The time step for metadynamics simulations is 2 fs, and the bias potential was added every 1000 steps. The grid bin for CV1 and CV2 was 1000. Each metadynamics simulation was run for 3 μs. The force, work, and bias were recorded every 100 ps, while the trajectories were saved every 200 ps. The free energy surface (FES) for each tracer was obtained by reweighting on the time-dependent bias.^59^ On FES, the energy (reweighted) difference between each site with a local minimum and solvent was used to roughly rank the detected sites.

### Molecular Dynamics Simulations

Molecular dynamics simulations were carried out, using Desmond,^60^ for the tracer binding sites identified by docking and metadynamics simulations. The OPLS3e force field^61^ was used for the protein and the tracers. The SPC solvent model^57^ was used to solvate the system. An orthorhombic shape of the box was chosen for simulation with a 10.0 Å buffering area. Counter ions were added to neutralize the system with the salt concentration of NaCl being 0.15 M. The Nose–Hoover chain and Martyna–Bobias–Klein methods are used for thermostat and barostat, respectively.^62,63^ Energy minimization using default settings was performed before production simulation. The systems were simulated for 100 ns in the *NPT* ensemble with the temperature and the pressure set at 300 K and 1 atm, respectively.

### MM/GBSA Calculations

The last 10 ns of the Desmond MD trajectories was used for MM/GBSA (molecular mechanics, the generalized Born model, and solvent accessibility) calculation using the Prime module in the Schrödinger Suite (version 2019-1).^64^ The OPLS3e force field was used to refine the complex with the continuum solvation model named VSGB (variable dielectric surface generalized Born).^65^ The residues within 10 Å of the ligand were included for minimization before MM/GBSA calculations. The mean values and deviations were calculated from the results of the extracted snapshots.

## Supporting Information Available

Figure S1 showing the workflow of this work; Figures S2 and S3 illustrating the convergence of the metadynamics simulations for CBD and AD tau, respectively; Figure S4 showing the changes of number of hydrogen bonds during the MD simulations for CBD2115-e1; Figure S5 showing how the metadynamics models were obtained; Figure S6 showing a comparison between our work and the experimental result; Figure S7 depicting the time evolution of RMSD for the metadynamics simulations; Figure S8 showing a binding free energy decomposition result for PI2620-e1; Tables S1 and S3 showing the approximated binding free energies from metadynamics simulations for the CBD and AD tau, respectively; Table S2 showing the docking scores of the tracers at E1, S3, and S14 sites (PDF)
