# Supplementary material for: Dissecting the Binding Profile of PET Tracers to Corticobasal Degeneration Tau Fibrils
Source: ACS Chem Neurosci. 2021 Aug 31;12(18):3487–96. doi: 10.1021/acschemneuro.1c00536 (PMC8447187; doi:10.1021/acschemneuro.1c00536)
Supplement: Supplementary file 1 — cn1c00536_si_001.pdf [file cn1c00536_si_001.pdf]

Supporting Information

Dissecting the Binding Profile of PET Tracers to Corticobasal Degeneration tau Fibrils

Yang Zhou[1,#], Junhao Li[1,#], Agneta Nordberg[2,3], Hans Ågren[1*]

[1] *Department of Physics and Astronomy, Uppsala University, Box 516, SE-751 20 Uppsala, Sweden*

[2] *Division of Clinical Geriatrics, Center for Alzheimer Research, Department of Neurobiology, Care Sciences and Society, Karolinska Institutet, 141 84, Stockholm, Sweden*

[3] *Theme Aging Karolinska University Hospital, S-141 86, Stockholm, Sweden*

[#] These authors contributed equally.

[*] Corresponding author: Hans Ågren

Email: hans.agren@physics.uu.se

Keywords: corticobasal degeneration; metadynamics; positron emission tomography tracer; free energy surface; Alzheimer disease; binding profile

This PDF file includes:

Figures S1 to S8
Tables S1 to S3

Figure S1. Illustration of the workflow implemented in this study.

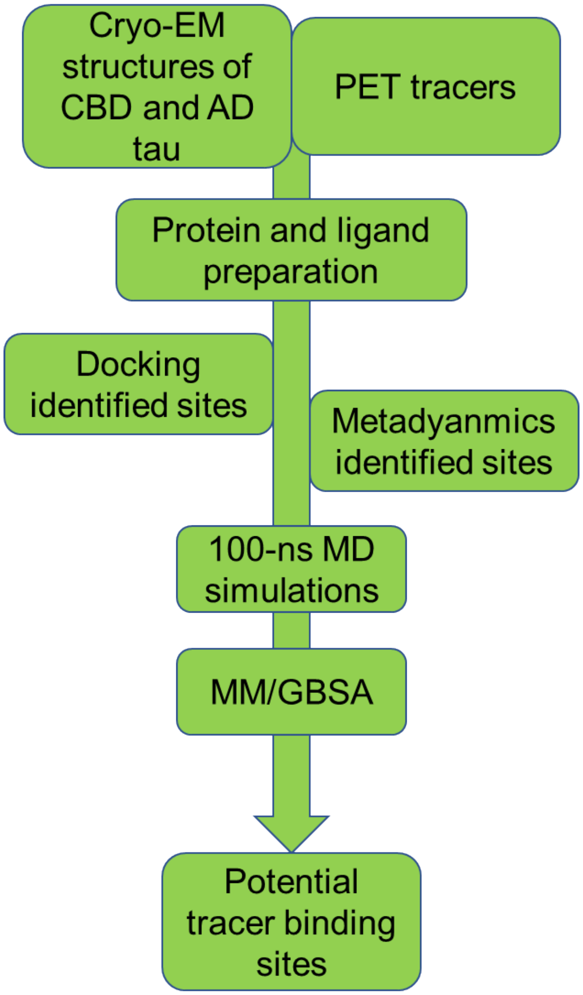

Figure S2. Illustration of convergence of the metadyanmics simulations for the CBD tau.

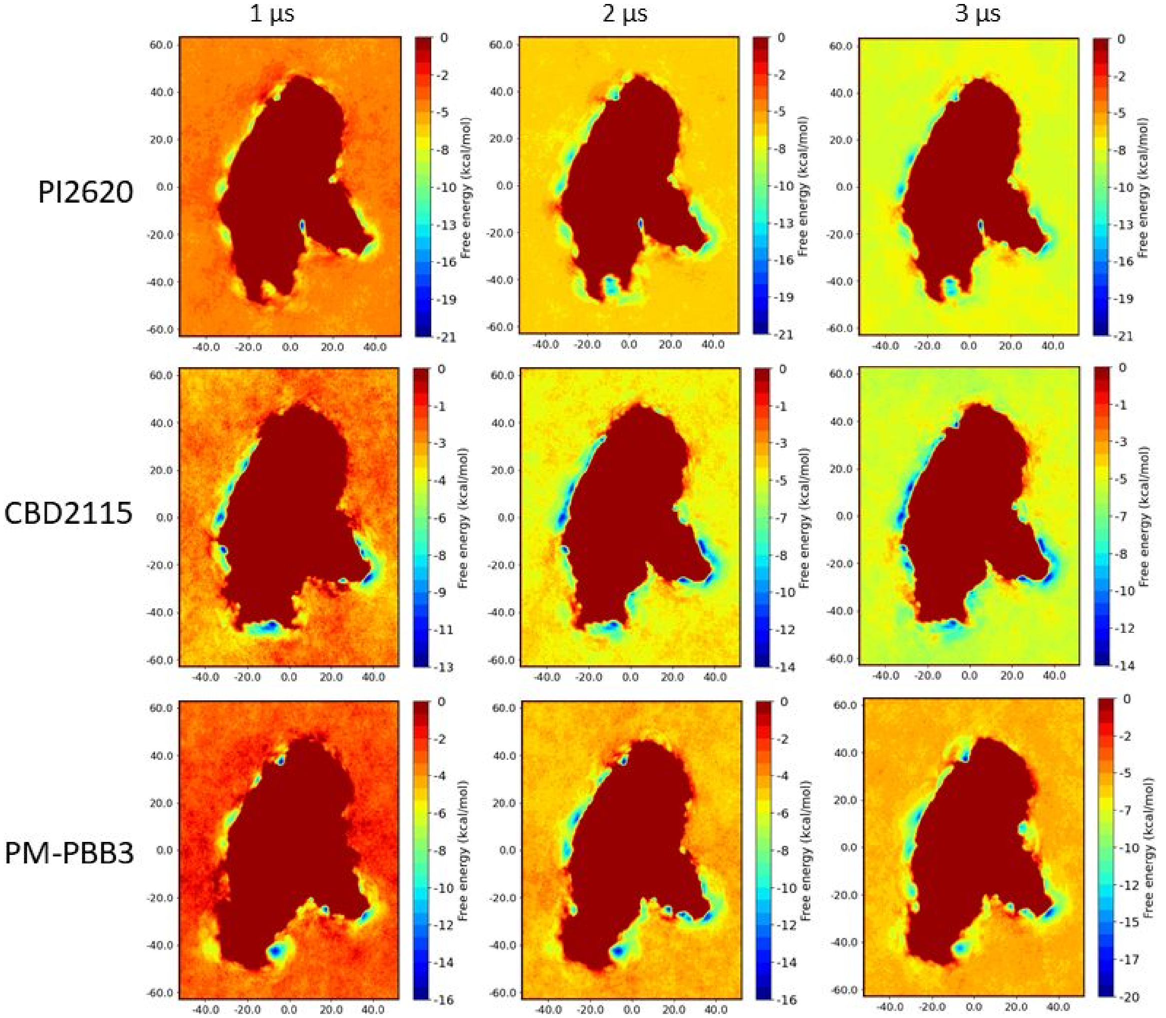

Between 2-3 μs, no new local minimums occurred on the surface, indicating that the simulation is converged for each system.

Figure S3. Illustration of convergence of the metadyanmics simulations for the AD tau.

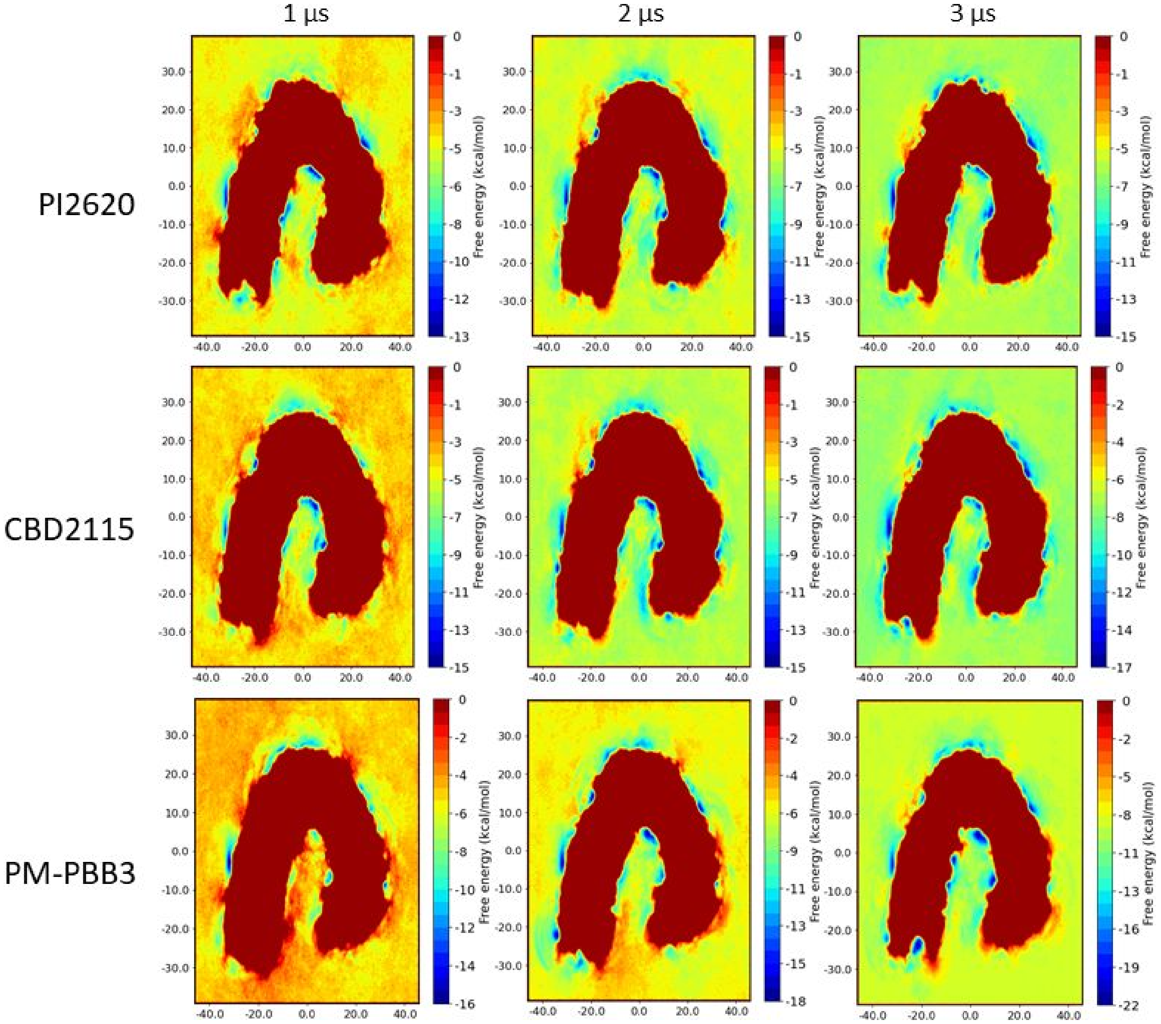

Between 2-3 μs, no new local minimums occurred on the surface, indicating that the simulation is converged for each system.

Figure S4. Time evolution of hydrogen bond number for MD simulations of CBD2115-e1 system

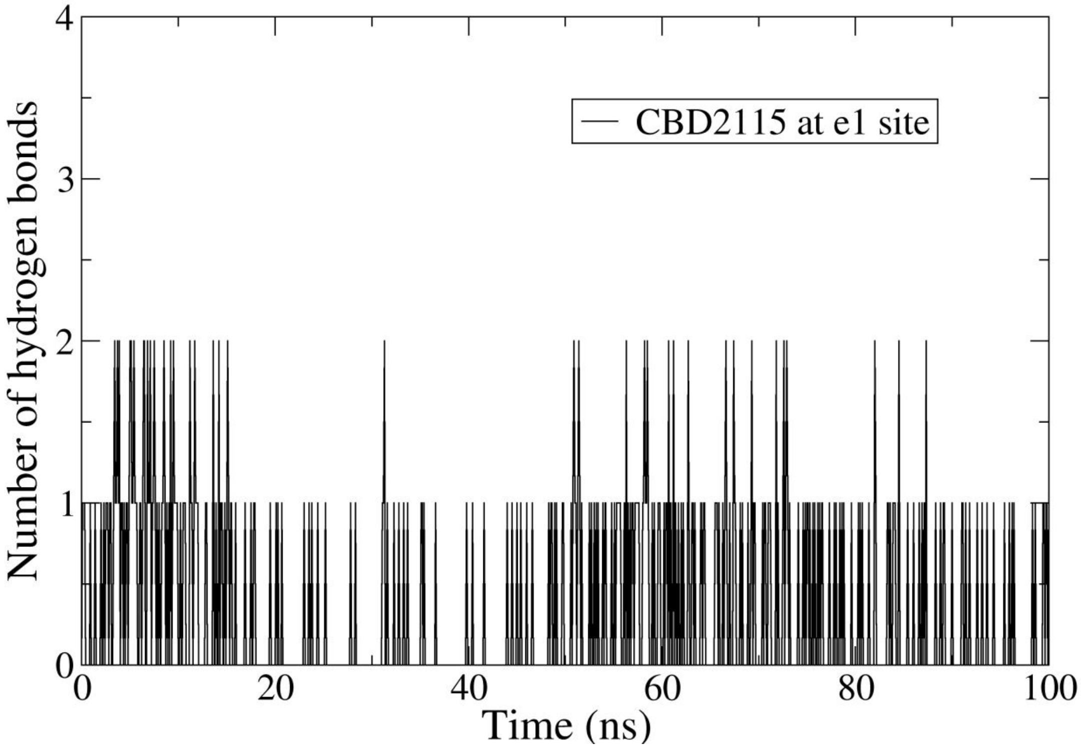

Figure S5. Illustration of the model used for metadynamics simulations and the collective variables selected.

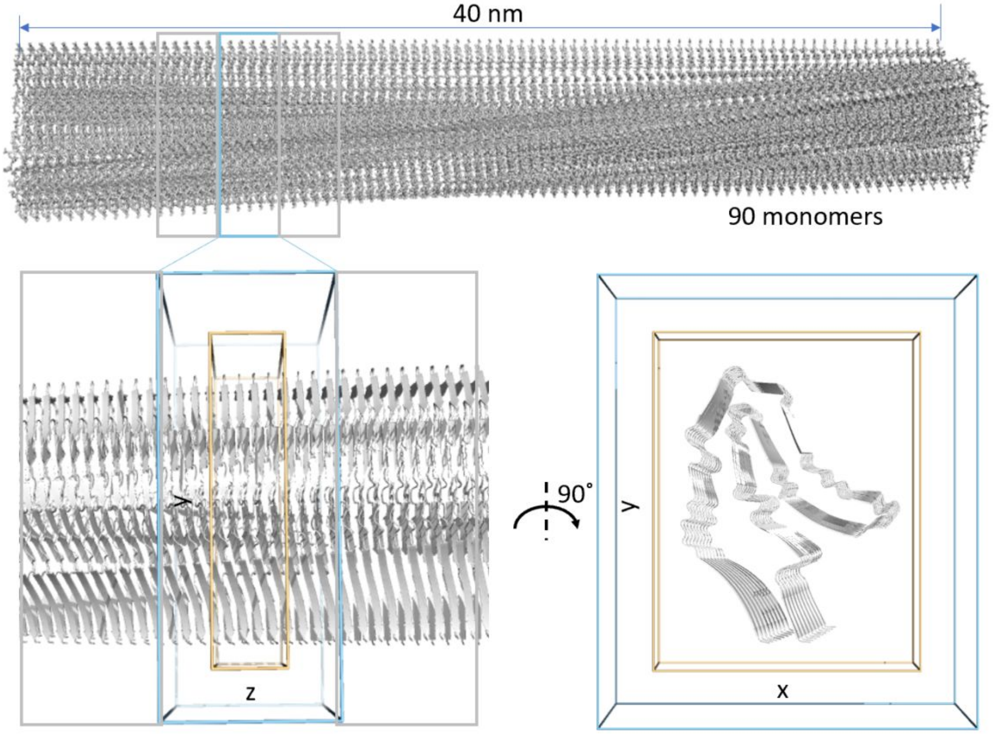

Figure S6. Illustration of the overlapping between experimental and theoretical models for identifying PM-PBB3 binding sites on AD tau.

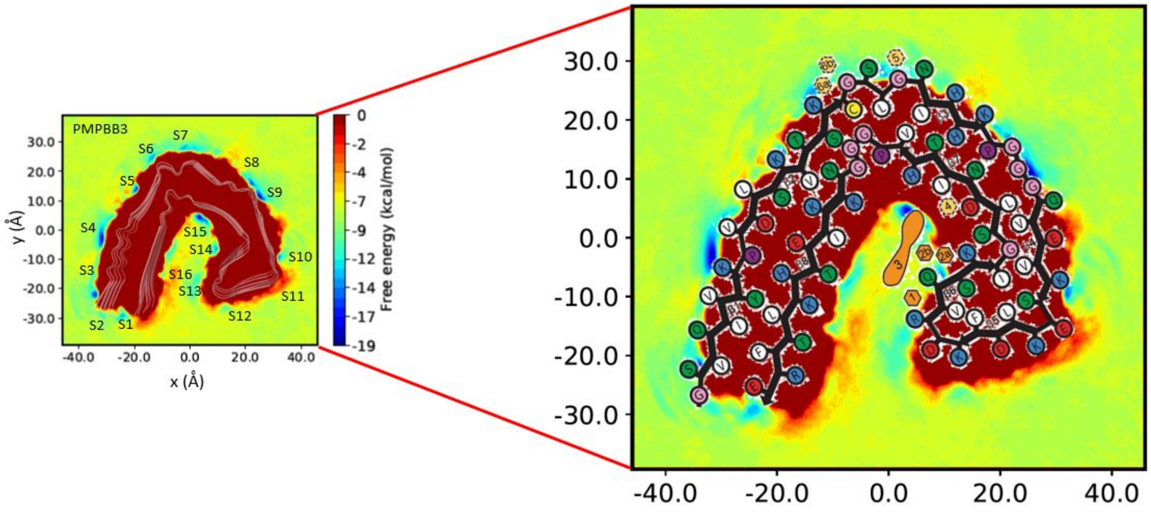

Superimpose of Fig. 1e in ref. 40 (reproduced from [*Acta Neuropathol.* ***2021***, *141, 697-708*]. Copyright. [2021] Springer Nature) and the Figure 4c in our study. The white pixels in the background of Fig. 1e have been replaced with transparent pixels for comparison.

Figure S7. Time evolution of protein Cα RMSD for the metadynamics simulations.

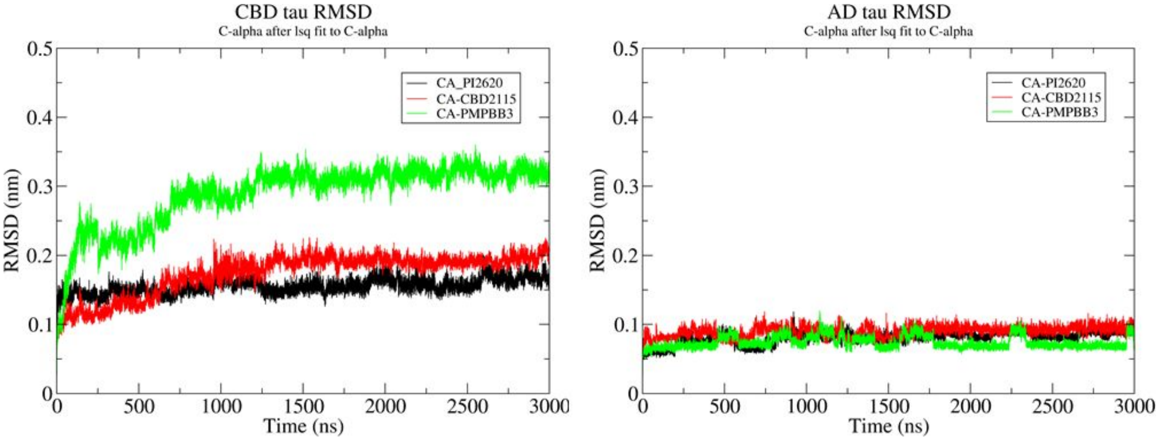

Figure S8. Binding free energy decomposition for PI2620 bound to the e1 site of CBD tau.

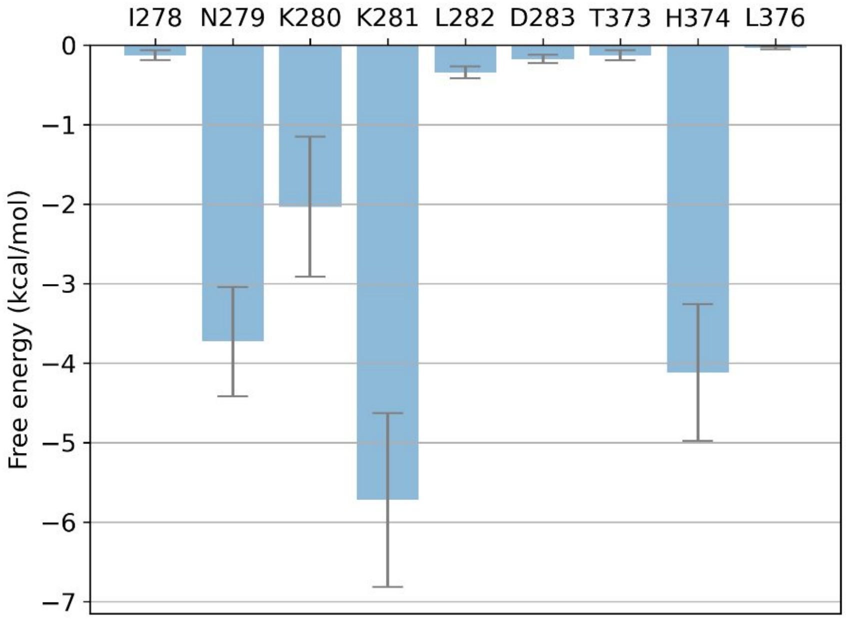

Table S1. Free energy differences for tracers binding to CBD tau from metadynamics simulations (kcal/mol).

Site	PI2620	CBD2115	PMPBB3
S1	**-7.5 ± 0.2**	-5.1 ± 0.1	**-7.1 ± 0.0**
S2	-6.4 ± 0.2	-5.7 ± 0.0	-6.0 ± 0.1
S3	-1.9 ± 0.1	-5.7 ± 0.2	-4.1 ± 0.0
S4	-3.8 ± 0.1	-5.4 ± 0.1	-5.3 ± 0.0
S5	-6.6 ± 0.0	**-7.7 ± 0.1**	**-7.8 ± 0.1**
S6	**-7.1 ± 0.1**	-6.3 ± 0.2	**-7.4 ± 0.2**
S7	-3.3 ± 0.2	-6.6 ± 0.1	-2.7 ± 0.1
S8	-6.3 ± 0.7	-6.6 ± 0.2	**-10.2 ± 0.1**
S9	-3.3 ± 0.1	-6.5 ± 0.1	-5.1 ± 0.2
S10	-6.1 ± 0.1	-6.5 ± 0.2	**-9.3 ± 0.2**
S11	-1.6 ± 0.0	-6.1 ± 0.0	-6.4 ± 0.2
S12	**-11.4 ± 0.4**	-	-1.4 ± 0.1

Table S2. Docking scores of the tracers at E1, S4, and S14 (kcal/mol).

	E1	S4	S14
MK6240	-9.2	-4.9	-6.0
PI2620	-8.8	-5.4	-6.5
CBD2115	-8.5	-6.4	-6.8
PMPBB3	-10.6	-4.8	-7.0

Table S3. Free energy differences for tracers binding to AD tau from metadynamics simulations (kcal/mol).

Site	PI2620	CBD2115	PMPBB3
S1	**-6.3 ± 0.1**	-0.7 ± 0.1	**-7.9 ± 0.2**
S2	-4.0 ± 0.2	-2.0 ± 0.1	**-**
S3	-4.8 ± 0.4	-3.8 ± 0.1	-4.0 ± 0.2
S4	-4.0 ± 0.3	**-7.9 ± 0.1**	**-6.8 ± 0.1**
S5	-5.6 ± 0.3	-3.7 ± 0.3	-5.2 ± 0.2
S6	-3.7 ± 0.1	-3.3 ± 0.2	-1.9 ± 0.2
S7	-3.2 ± 0.5	-3.9 ± 0.1	-4.7 ± 0.0
S8	-4.2 ± 0.1	-	-3.0 ± 0.4
S9	-5.2 ± 0.3	-5.7 ± 0.1	**-6.7 ± 0.1**
S10	-5.3 ± 0.1	-4.4 ± 0.1	-
S11	-4.1 ± 0.3	**-6.7 ± 0.1**	**-**
S12	-3.5 ± 0.1	-1.2 ± 0.2	-
S13	-5.6 ± 0.1	-3.7 ± 0.1	-1.0 ± 0.1
S14	-5.0 ± 0.1	-5.4 ± 0.2	**-6.5 ± 0.2**
S15	**-6.5 ± 0.1**	**-6.2 ± 0.1**	**-7.6 ± 0.1**
S16	-4.6 ± 0.1	-5.0 ± 0.1	-4.7 ± 0.2
